# Development and Evaluation of a Pillow to Prevent Snoring Using the Cervical Spine Recurve Method

**DOI:** 10.1155/2022/2561107

**Published:** 2022-08-17

**Authors:** Dohyun Ahn, Hyeunwoo Choi, Jongmin Lee, Sung-Phil Heo

**Affiliations:** ^1^Department of Medical & Biology Engineering, Kyungpook National University, Daegu 41566, Republic of Korea; ^2^Molecular Hemodynamic & Computational Laboratory, Daegu 41566, Republic of Korea; ^3^Kyungpook National University Hospital, Daegu 41944, Republic of Korea; ^4^Department of Information and Communication Engineering, Gangneung-Wonju National University, Gangneung-si 25457, Republic of Korea

## Abstract

Snoring lowers the quality of sleep, causing many secondary diseases. In response, various types of preventive devices were manufactured, but most of them were far from actual snoring prevention by only sensing snoring and giving feedback. In this study, we proposed a new method to prevent snoring by adjusting the posture during sleep by widening the oropharynx space. An increase in the oropharynx area was confirmed through the expansion of the cervical spine, and a dedicated pillow that can extend through an angle of up to 20° was manufactured. Through this developed method, it was possible to easily extend the cervical spine angle in a supine position to the user, and the frequency of snoring was then tested. As a result, it was confirmed that by using the pillow with an expansion angle of 20° or more, snoring did not occur. Furthermore, looking at the evaluation results of the subjective levels of satisfaction, sleep-related items received an average of 5.9 or higher, and function-related items received high scores with an average of 5.7. We can confirm that the reliability of performance evaluation will be dramatically improved if the scope of the subject group is expanded to include various body types, ages, and genders and conduct performance evaluations for each group.

## 1. Introduction

**Figure 1:**
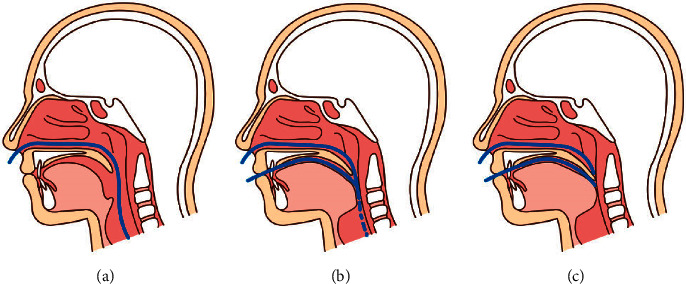
Causes and processes of snoring and sleep apnea: (a) normal, (b) oropharynx narrowed when snoring, and (c) nearly obstructed airways during sleep apnea [1].

As can be seen in Figure 1, snoring is only a symptom that indicates narrowing of the airway. Breathing is a typical process where the diaphragm contracts and the lungs expand like balloons, causing air to be sucked in. If there is a narrowed area in the airway between the nose entrance and the lungs, the phenomenon where the fluid area vibrates and makes a sound due to the rapid flow of air is called snoring. When the nose is severely blocked, the person breathes through the mouth, the chin drops back, and the back of the tongue is blocked, which also causes breathing difficulties. Studies have proven that snoring increases and sleep apnea occurs when a normal person sleeps with their nose covered. Adenoid hypertrophy is the most common cause of narrowing or clogging of the airway behind the nose in children and adolescents [2].Even people with narrowed airways do not snore when they are awake, but only when they sleep. The big difference between waking and sleeping is muscle tension. As you wake up, all the muscles of the body become tense, and even if the airflow is fast through breathing, there is no vibration phenomenon. However, when you fall asleep, muscle tension is released, and vibration occurs easily.

**Figure 2:**
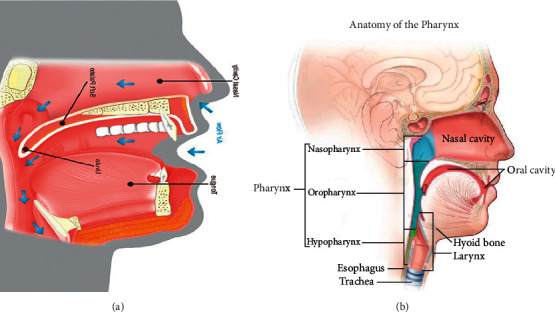
Air flow during sleep and anatomy of the pharynx: (a) air flow during sleep and (b) anatomy of the pharynx [3].

Additionally, through weight gain or when the mucous membrane swells, the oropharynx part of the pharynx (shown in Figure 2) going to the upper airway narrows, which is one of the important factors of snoring [4–17].

In addition, in previous studies, it was confirmed that the cause of OSA (obstructive sleep apnea) was caused by osteophytes of the cervical spine. Anterior osteophytes in the cervical vertebrae cause snoring and dyspnea, and the use of a pillow may be considered to overcome them nonsurgically, and additional research is needed on this. In addition to the existing pharyngeal fat pad area and the long distance from the cervical spine to the hyoid bone, it was confirmed that the short distance from the mandibular symphyseal to the cervical vertebrae increased the risk for OSA. However, there were no additional studies on the improvement of snoring according to the area change of the oropharynx.

Pillows can be used to prevent snoring. Pillows are bedding that maintain a comfortable sleeping posture by supporting the neck (cervical spine) for movements occurring during sleep, and such research has been conducted on their height and texture. The aspects of the pillow that are important for sleep include thermal conductivity, height, size, elasticity, shape stability, hygroscopicity, and air permeability. It was found that the height of the pillow had a significant effect on sleep [18]. The height of the pillow is closely related to the angle of the cervical spine that appears when sleeping. It is said that high pillows interfere with cervical curvature by increasing cervical spine angles, cause blood circulation disorders due to veins in the neck area, cervical abnormalities or tension in neck muscles, and even result in cerebral hemorrhage or stroke during long-term use [19]. When the neck is bent, the intervertebral nerves passing between the cervical vertebrae are compressed, resulting in pain. Therefore, the height of the pillow should be designed to maintain a natural neck angle to facilitate blood circulation and relieve nerve pressure. Recently, pillow products with various shapes and materials, such as buckwheat, memory foam, and latex, have been released and were recognized for their functionality. However, the design of these pillows is based only on the lying position among sleeping positions, and the effect of angle adjustment is not considered [20]. The study aimed to test whether snoring could be prevented by adjusting the posture during sleep by widening the oropharynx space (the angle between the body of the mandible and cervical vertebrae No.7) by using a specially designed pillow.

## 2. Materials and Methods

### 2.1. Setting the Standard for the Expansion Angle of the Cervical Vertebrae

First, in order to observe oropharynx, a cervical X-ray was taken for three volunteers (IRB: GWNUIRB-2020-30).

**Figure 3:**
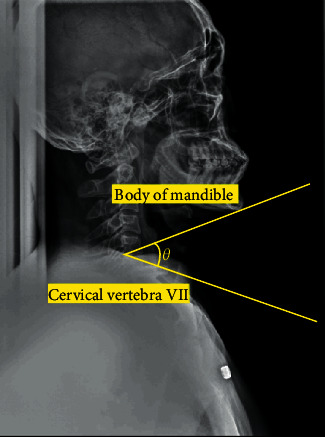
The angle formed by the body of the mandible and the cervical vertebra VII (No.7).

**Figure 4:**
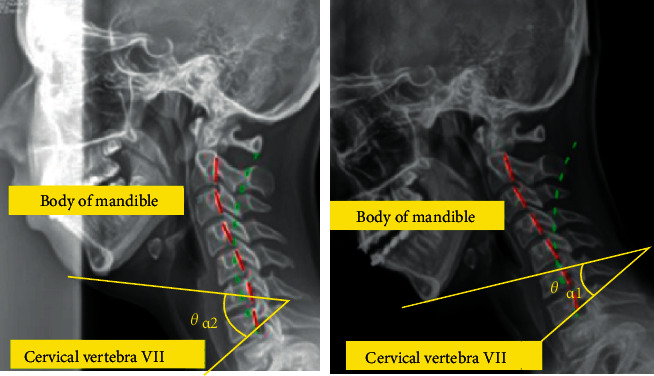
Experiments for measuring the angle of expansion.

Then, to confirm the increase in the area of the oropharynx, an X-ray image of the head was obtained. As shown in Figure 3, the angle between the mandible body at the end of the jaw and the part just above the cervical vertebra No. 7 at the end of the cervical spine was set as the standard for expansion. Through this, the degree to which the angle of the neck is expanded can be confirmed. Before the expansion experiment, in the case of anteflexion, the amount of planar change in the oropharynx was measured. From Figure 4, it was confirmed that the reduction angle was 5° if *Ɵ*_a1_ of *b* (anteflexion posture) was subtracted from *Ɵ*_a2_ of a (basic posture) and as a result of calculating the area of the oropharynx, it was confirmed that it decreased by 233 mm^2^.

Additionally, when the angle between the mandible body at the end of the jaw and the part just above the cervical vertebra No.7 was maximally expanded, the expansion angle was about 30°, and in the case of 1° to 4° within the same expansion section, the increase in the area was insignificant; however, the number of samples increased, confirming that it was inefficient and it was intended to be measured in 5° units with a difference of at least 50 mm^2^ in the plan area.

### 2.2. Oropharynx X-Ray Image Acquisition According to the Angle

**Figure 5:**
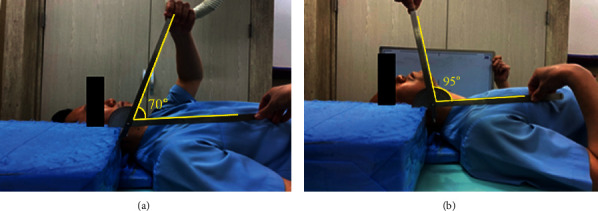
Experiments for measuring the angle of expansion: (a) supine position and (b) posture when the cervical spine is fully extended.

To determine the level of angle between the mandible body at the end of the jaw and the part just above the cervical vertebra No.7, the KOISS's KL-3DG laser leveler was used. Thus, the posture in which the subject lay down and looked straight up was designated as a reference point. As can be seen in Figure 5, an X-ray was taken while the subject had the head extended backward from the reference point at an angle of 5° ±0.1 over 6° and extended to the point where the pain was felt.

### 2.3. Calculation of Oropharynx Area According to the Angle

**Figure 6:**
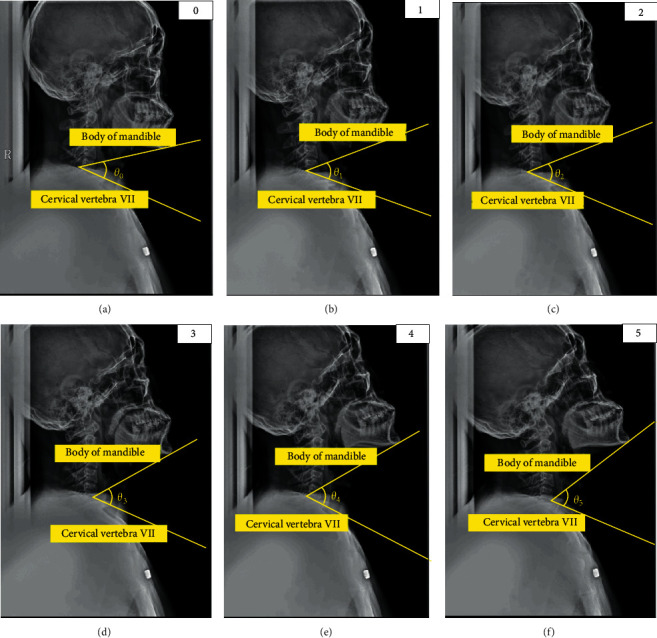
Five oropharynx area X-ray images obtained at random angles: (a) origin point (*θ*_0_), (b) 5.03° expansion (*θ*_1_) from *θ*_0_, (c) 5.01° expansion (*θ*_2_) from *θ*_1_,(d) 4.95° expansion (*θ*_3_) from *θ*_2_, (e) 4.99° expansion (*θ*_4_) from *θ*_3_, and (f) 5.02° expansion (*θ*_5_) from *θ*_4_.

The X-ray images of six angles were obtained (shown in Figure 6), and the angles from the origin point were measured.

**Figure 7:**
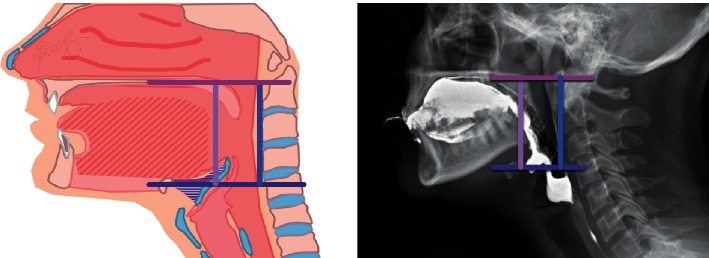
Oropharynx [21].

First, when lying in the right position, the angle formed by the body of the mandible and cervical vertebrae No. 7 at the end of the cervical spine was calculated, and the expansion angle was estimated based on the angle. Based on the information in Figures 2 and 7, the area of the oropharynx was specified by two experts [22] as a space from the C1 vertebral process to the hyoid bone, and the area was calculated using the ImageJ program [23]. Looking at the increase compared to the origin, it was confirmed that the area of the oropharynx was increased 1.54 times at 14.9° in Step 3, and that when the final 25° was reached, the area was expanded by a factor of 1.72 times (shown in Table 1).

### 2.4. Derivation of the Pillow Application Angle

**Figure 8:**
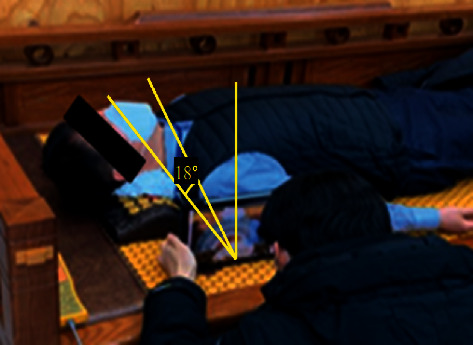
Angle derivation experiment that feels uncomfortable when extending the angle.

The area of the oropharynx, according to the expansion of the angle between the body of the mandible and cervical vertebrae No.7, was identified. To calculate the angle of pain during expansion, 33 men and women (the subjects listened to the explanation of the purpose of the experiment and the measurement method and agreed) in their 20s and 50s were tested at the angle of pain when extending between the body of the mandible and cervical vertebrae No.7 (shown in Figure 8).

As a result of the experiment (shown in Table 2), it was confirmed that the male group felt uncomfortable from 18.3° on an average, and the female group felt pain in the neck area from an average of 22.2°. The overall average was confirmed to be 20°. Therefore, it was decided to manufacture the pillow to give an expansion angle of up to 20°, and the pillow model was devised to give an angle rotation in turn.

### 2.5. Production of Angle-Extended Pillows

Pillows (3H-HN1) are designed with a stop for each angle and their angles can be up to 20°.

**Figure 9:**
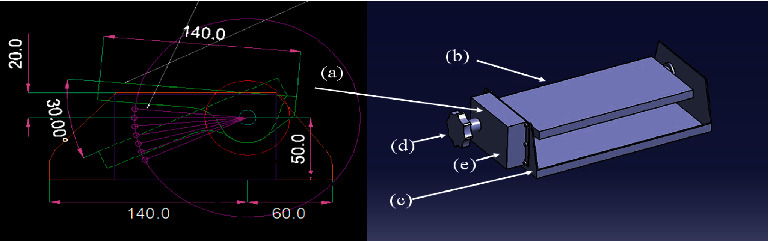
The operating mechanism and conceptual diagram of the proposed pillow.

**Figure 10:**
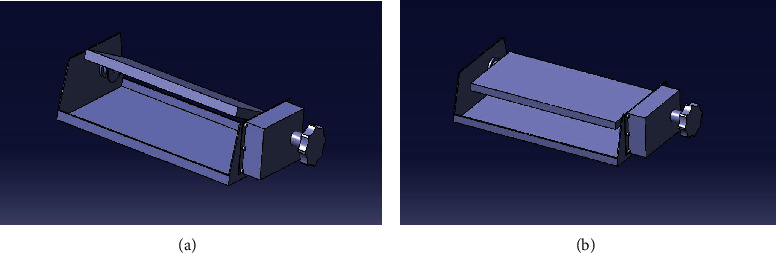
A 3D drawing of the proposed pillow's operating mechanism.

In Figure 9, (a) is configured to give an expansion angle of 20° by holes punched at intervals of 5°, when adjusting the angle, a ball slots into the perforated hole and is designed to be stationary; (b) forms a pillow support by bending an aluminum plate 2T (mm); (c) is part of the frame and has an aluminum plate thickness of 3 T (mm); (d) is an angle adjustment unit; and (e) is the housing for the angle adjustment device (shown in Figure 10).

The body of the unit was manufactured according to the design drawing, a sponge was inserted to smoothly support the head, and lastly, an angle adjustment pillow was manufactured [24].

**Figure 11:**
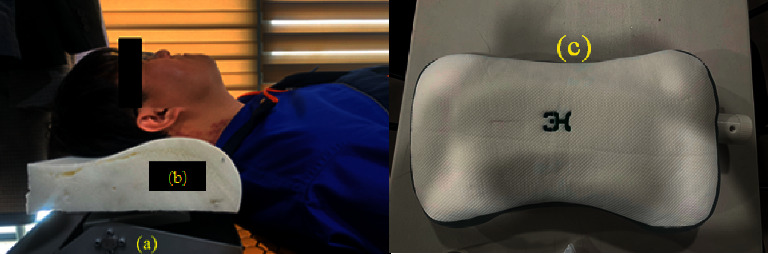
Prototype of proposed pillow.

In Figure 11, (a) is an operating unit capable of giving an angle and (b) is a sponge that supports the head. By combining them, the pillow was made as shown in (c).

### 2.6. Method of Evaluation

The study aimed to test whether the angle between the body of the mandible and cervical vertebrae No.7 that ends at the cervical spine could be enlarged through a pillow and whether this could alleviate snoring. First, to check whether it was effective for snoring, five men who snore were tested to monitor for snoring for 30 min, while sleeping on the pillow at an extended angle of 20°. At this time, the participants were those who had snoring as a result of previous observation but did not receive treatment because they did not recognize it as a disease, and those with cervical spine disease were excluded. In addition, the number of patients was selected after actively reviewing whether the patient would have a negative effect due to hyperextension of the spine, and there was no risk of injury. In addition, in the previous study, it was confirmed that the effect of preventing snoring when lying on the side more than 30° was confirmed; so, in this paper, the method of lying on the side and sleeping on a pillow was excluded [25].

A natural latex pillow of the same size was provided, which had the same height as the angle expansion pillow; however, it did not have an angle expansion function, and snoring was checked for 30 min.

The subjects listened to the explanation of the purpose of the experiment and the measurement method and agreed to the experiment. The subject's height and weight were measured by direct measurement according to KS ISO7250-11 [26] standard, the Basic Human Body Measurements for Technological Design of Korea Standard. After taking a break for about five minutes, the experiment was conducted after confirming that the subjects had entered the sleep for state in a position lying on the bed (in a posture looking at the ceiling) with the pillow. The subjects' height, weight, and BMI are shown in Table 3.

Second, the subjects evaluated their subjective satisfaction with the comfort of the pillow before and after use in writing as shown in Table 4. The total number of respondents was five. The question that directly checks the subjective satisfaction part was evaluated as points. Overall subjective satisfaction was evaluated after the experiment was conducted, by each subject filling out a Likert scale-based questionnaire to maintain consistency [27]. The subjective evaluation items were classified into discomfort and sleep quality and rated on the scale as shown in Table 5, with 1 being “strongly disagree,” 4 being “neutral”, and 7 being “strongly agree.”

## 3. Results and Discussion

**Figure 12:**
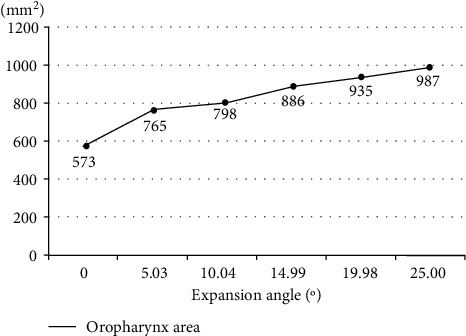
Increase in flat area versus expansion angle.

In Figure 12, the result of the increase in the area of the oropharynx due to the cervical spine recurve is shown. It was confirmed that the larger the expansion angle according to the cervical spine recurve, the linear the increase in the planar area of the oropharynx.

### 3.1. Results of the Snoring Experiment

Table 6 shows when comparing the angle expansion pillow developed in this study with the standard pillow (Latex pillow of the same height as the pillow developed in research) for the five test subjects, snoring did not occur for 30 min of sleep with the angle expansion pillow; however, it did occur at least five times on average with the standard pillow (Latex pillow of the same height as the pillow developed in research). In addition, movement during sleep was observed five test subjects, but no special movement was observed, and no one escaped from the pillow during sleep. When comparing the angle expansion pillow developed in this study with the standard pillow (latex pillow of the same height as the pillow developed in research) for the five test subjects, snoring did not occur for 30 min of sleep with the angle expansion pillow; however, it did occur at least five times on average with the standard pillow (latex pillow of the same height as the pillow developed in research). In addition, movement during sleep was observed in five test subjects, but no special movement was observed, and no one escaped from the pillow during sleep.

**Figure 13:**
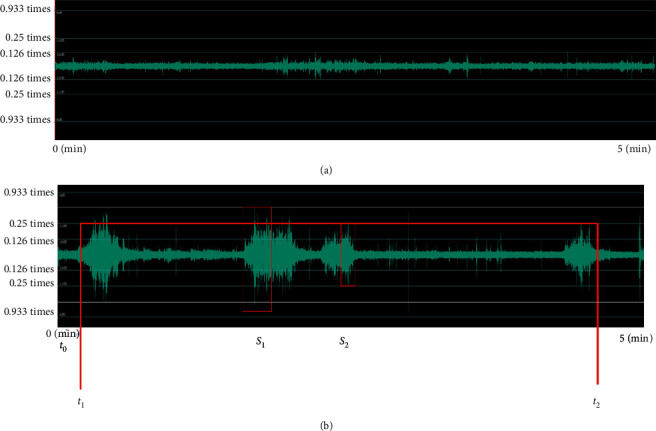
Voice waveform during experiment: (a) when an expansion angle of more than 20° is given, (b) standard pillow: latex pillow of the same height as the pillow developed in research.

In Figure 13(a) shows a negative waveform for five minutes when the proposed angle expansion pillow was used, and it can be confirmed that there was no snoring. and Figure 13(b) shows a negative waveform in the five minute section when a standard pillow (latex pillow of the same height as the pillow developed in research) was used. Snoring started at the t_1 section (*t*_1_) in the first t_0 (*t*_0_) to 15 seconds and occurred four times until the 4 min and 16 sec section t_2 (*t*_2_). The amplitude was confirmed from a minimum of 0.25 times (S_2) to a maximum of 0.355 times (S_1). This measured result can be said to be higher than the maximum amplitude of external noise of 0.25 times.

### 3.2. Evaluation Results

Table 7 shows the results of the functional evaluation (average score of 5 subjects) of the proposed pillow. First, sleep-related aspects received high scores with sleep satisfaction (I-1) at 6.0 points, tiredness recovery (I-2) at 6.2 points, and degree of sleep-friendliness (I-3) at 5.4 points. The functional aspects received high scores and were rated as follows, possibility of posture adjustment (II-1) at 5.4 points, convenience of use (II-2) at 6.4 points, and pillow size (II-3) at 5.2 points.

## 4. Conclusion

In this study, we proposed a new method to prevent snoring by adjusting the posture during sleep by widening the oropharynx space. An increase in the oropharynx area was confirmed through the expansion of the cervical spine, and a dedicated pillow that can extend through an angle of up to 20° was manufactured. Through this, it was possible to easily extend the cervical spine angle in a supine position to the user, and the frequency of snoring was then tested. As a result, it was confirmed that through using the pillow with an expansion angle of 20° or larger, snoring did not occur. Furthermore, looking at the evaluation results of the subjective levels of satisfaction, a Likert scale was composed and applied with a total of seven levels, with 1 being “strongly disagree,” 4 being “neutral”, or 7 being “strongly agree.” Sleep-related items received an average of 5.9 or higher, and function-related items received high scores with an average of 5.7. However, it is necessary to conduct further experiments with the user's bio-signals to measure more objective satisfaction. We can confirm that the reliability of performance evaluation will be improved if the scope of the subject group is expanded to include various body types, ages, and genders and conduct performance evaluations for each group. In addition, in future studies, long-term follow-up studies will be conducted through VAS or ODI, such as pain that may appear due to hyperextension in the long-term use of pillows.

Additionally, only snoring was monitored in the experiment, but in the future, it will be necessary to obtain more objective results by performing bio-signal analysis, such as the respiratory rate [28, 29].

## Figures and Tables

**Table 1:** Oropharynx area according to the angle of expansion.

Location	Expansion angle (°)	The angle formed by the body of the mandible and the cervical vertebra No. 7 (°)	Oropharynx area (mm^2^)	Increase compared to origin (times)
Origin point (*θ*_0_)	0	35.0	573	0
1 (*θ*_1_)	5.03	40.0	765	1.33
2 (*θ*_2_)	5.01	45.0	798	1.39
3 (*θ*_3_)	4.95	50.0	886	1.54
4 (*θ*_4_)	4.99	55.0	935	1.63
5 (*θ*_5_)	5.02	60.0	987	1.72

**Table 2:** Angle that feels uncomfortable depending on the angle of expansion.

Group	Mean ± SD
Male	18.31° ± 7.34°
Female	22.28° ± 11.45°
All	20.00° ± 9.35°

**Table 3:** Subject physical condition.

Index	Mean ± SD
Height (cm)	175.6 ± 4.9
Weight (kg)	84.8 ± 4.2
BMI (kg/m^2^)	27.4 ± 0.1

The laboratory temperature (25.0 ± 2.0) °C, humidity (50.0 ± 5.0) were fixed.

**Table 4:** Satisfaction Evaluation with pillow.

Classification		Evaluation item
Sleep satisfaction	I-1	How satisfied were you with your sleep when using a pillow?
	I-2	Did you feel you recovered from your tiredness?
	I-3	Did the pillow induce sleep?

Functional satisfaction	II-1	Were you able to adjust your posture easily?
	II-2	Was it convenient to use?
	II-3	Are the pillows the right size?

**Table 5:** Likert scale table used in this experiment.

Response	Strongly disagree	Disagree	Slightly disagree	Neutral	Slightly agree	Agree	Strongly agree
Scale	1	2	3	4	5	6	7

**Table 6:** Rate of snoring when using the suggested pillow and normal pillow during sleep.

Classification	Mean ± SD
The rate of snoring in suggested pillow	0 ± 0
The rate of snoring in standard pillows	14.8 ± 8.9

※ standard pillow: Latex pillow of the same height as the pillow developed in research.

**Table 7:** The result of satisfaction evaluation with a pillow.

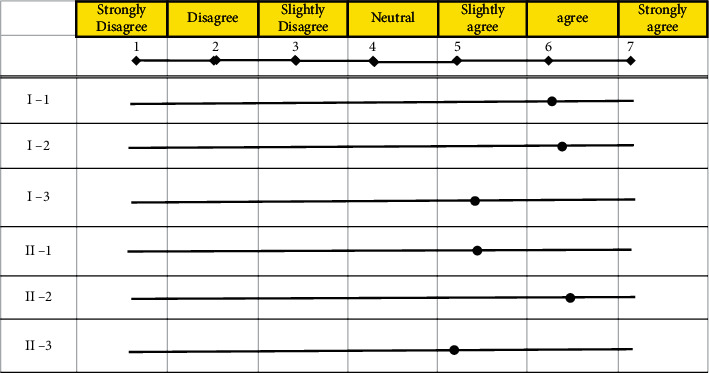

## Data Availability

The data that support the findings of this study are available from the first author (tigerace5012@nate.com) upon reasonable request.
